# Mendelian randomization study reveals a causal relationship between rheumatoid arthritis and risk for pre-eclampsia

**DOI:** 10.3389/fimmu.2022.1080980

**Published:** 2022-12-12

**Authors:** Dingyi Zhang, Ying Hu, Weijie Guo, Yang Song, Lin Yang, Shuhan Yang, Taoaixin Ou, Yanxu Liu, Yaoyao Zhang

**Affiliations:** ^1^ Department of Obstetrics and Gynecology, Key Laboratory of Birth Defects and Related of Women and Children of Ministry of Education, West China Second University Hospital, Sichuan University, Chengdu, Sichuan, China; ^2^ West China School of Medicine, Sichuan University, Chengdu, China; ^3^ Reproductive Endocrinology and Regulation Laboratory, West China Second University Hospital, Sichuan University, Chengdu, China; ^4^ Department of Pharmacy Services Tacoma, St. Joseph Medical Center, Catholic Health Initiative (CHI) Franciscan Health System, Tacoma, WA, United States; ^5^ Department of Pharmacy, West China Hospital, Sichuan University, Chengdu, Sichuan, China

**Keywords:** rheumatoid arthritis, pre-eclampsia, pregnancy, causal effect, mendelian randomization, genome-wide association studies

## Abstract

**Background:**

Epidemiological observational studies have investigated the relationship between rheumatoid arthritis(RA) and pre-eclampsia, but no consistent conclusions were obtained due to various limitations. Hence, we conducted a two-sample mendelian randomization analysis to evaluate the potential causal effect of RA on pre-eclampsia.

**Methods:**

Summary-level statistics for RA were derived from a large-scale meta-analysis of datasets of genome-wide association studies(GWAS) which involved 14,361 cases and 43,923 controls. Moreover, summary statistics for pre-eclampsia or eclampsia were sourced from the Finn biobank which contained 3,903 cases and 114,735 controls. The inverse variance weighting (IVW) as well as other four effective methods including MR-Egger, weighted median, weighted mode, and simple mode were applied to deduce the potential causal relationships between RA and pre-eclampsia comprehensively.

**Results:**

The two-sample MR analysis suggested a strong causal relationship between RA and pre-eclampsia[OR,1.05;95%CI, 1.01-1.09;p<0.05]. The OR estimates obtained from the weighted mode[OR,1.09;95%CI,1.03-1.15;p<0.01] and weighted median[OR,1.07;95%CI, 1.01-1.14;p<0.05] were similar to those from the IVW method, but there was no significant association observed in MR Egger and simple mode analysis.

**Conclusion:**

This MR analysis provides evidence of a positive causal association between RA and pre-eclampsia genetically. Our findings highlight the importance of more intensive prenatal care and early intervention among pregnant women with RA to prevent potential adverse obstetric outcomes. Moreover, our study provides clues for risk factor identification and early prediction of pre-eclampsia.

## Introduction

Hypertensive disorders of pregnancy(HDP) are common pregnancy complications, which are comprised of gestational hypertension, pre-eclampsia or eclampsia, pre-eclampsia superimposed on chronic hypertension, and chronic hypertension. HDP, especially pre-eclampsia, complicate 2-8% of pregnancies and account for a substantial proportion of maternal and perinatal mortality (1, 2). Worldwide, more than 50,000 pregnant women and 500,000 fetuses have died from pre-eclampsia (3). The multifactorial pathogenesis of HDP is complex, and it can be influenced by the environment during pregnancy as well as the underlying pathological and immunological conditions of patients. Pre-eclampsia as a type of HDP, results from heterogeneous causes (4).Some potential risk factors for pre-eclampsia include history of pre-eclampsia, chronic hypertension, pre-gestational diabetes, multifetal pregnancy, obstetric complications in a previous pregnancy such as fetal growth restriction, stillbirth, abruption, and autoimmune diseases including antiphospholipid syndrome and systemic lupus erythematosus (5, 6). However, most of factors that have been identified lack accuracy in predicting its onset and preventative therapies only moderately reduce a woman’s risk of pre-eclampsia. Therefore, it is crucial to identify more reliable risk factors for the identification and early prediction of pre-eclampsia.

Rheumatoid arthritis(RA) is a systemic autoimmune disease with a chronic inflammatory process, which has a female predominance (7). Several studies have investigated the potential relationship between RA and pre-eclampsia and provided conflicting results, possibly due to residual confounding. A cohort study of 312,081 women has reported that women with RA were significantly more likely to have hypertensive disorders in pregnancy [OR,1.51;95% CI, 1.16-1.97;p<0.01] (8). Particularly for pre-eclampsia, a nationwide population-based study in Taiwan showed that the odds of women with RA suffering from pre-eclampsia was 2.22 times that of women without RA[OR,2.22; 95%CI, 1.59-3.11] (9). However, another research showed that although the confidence intervals approached significance for outcomes, no greater risk existed among women with RA than non-RA women of suffering from pre-eclampsia[aRR,1.08;95% CI, 0.97- 2.50] (10). Owing to the complication of pregnancy, and study deficiencies such as geographical and ethnic differences, reverse causation, selection bias, and other potential biases (11), these observational studies do not adequately and directly reflect the causal association between RA and pre-eclampsia.

Mendelian randomization(MR) is an emerging analysis method that uses genetic variants as instrumental variables (IVs) to evaluate the causal effect of modifiable exposures on outcomes (12). Because of the randomization of genetic variant inheritance, MR analysis can reduce the possibility of being influenced by potential confounders and reverse causation bias, inferring the correlation between exposure and outcome genetically, thus providing more reliable results (13). Therefore, we conducted a two-sample MR analysis to explore the causal effect of RA on pre-eclampsia.

## Method

### Study design

**Figure 1:**
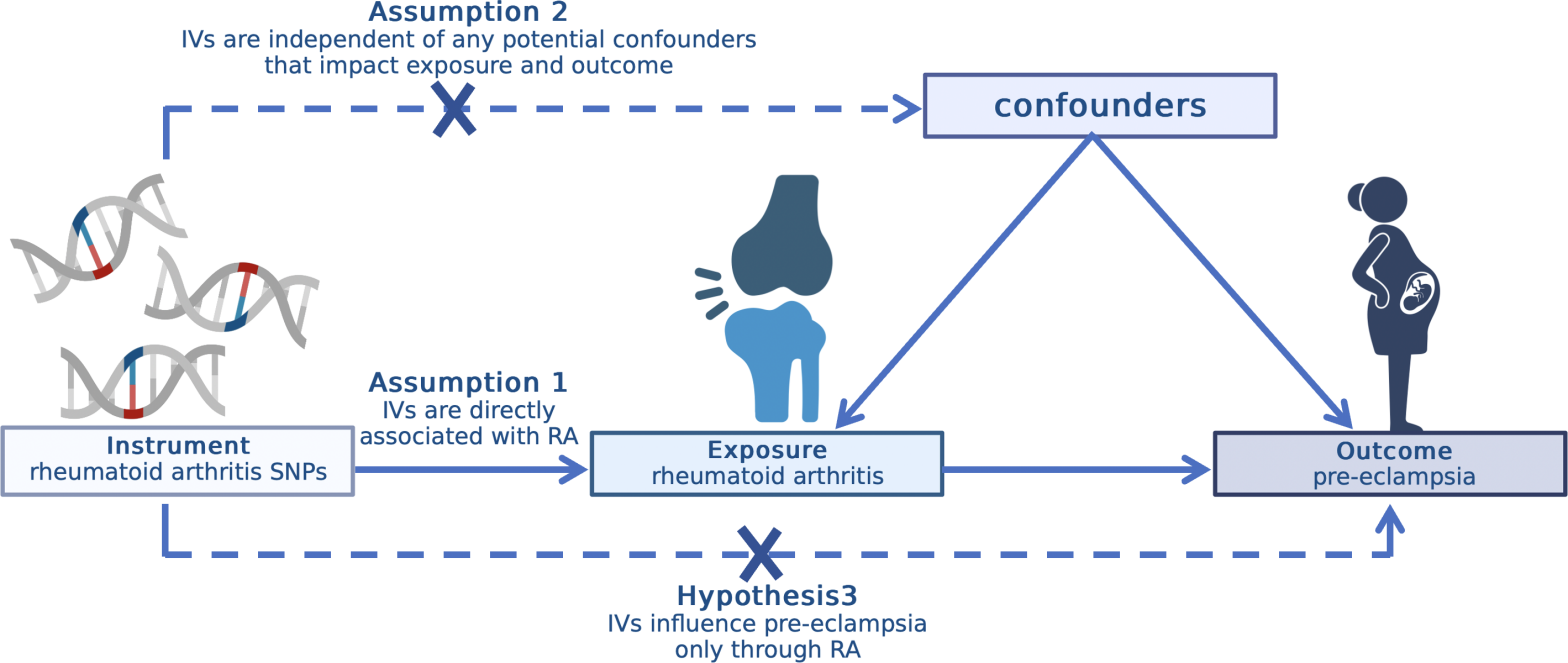
Overview of the MR design. MR, mendelian randomization.

In this study, we conducted a two-sample MR to evaluate the causal association between RA and pre-eclampsia. SNPs were used as instrumental variables (11). To maximize the accuracy of results, three important hypotheses must be confirmed during the whole process (14). First, the selected IVs should be directly associated with RA. Second, the IVs are independent of any potential confounders that impact exposure and outcome. Third, the IVs influence pre-eclampsia only through RA. All original studies acquired ethical review approval and informed consent (**Figure 1**).

### Data source

Datasets of genome-wide association studies(GWAS) provide reliable instruments for MR analyses. In our study, summary-level statistics for RA were derived from a large-scale meta-analysis of GWAS which involved 14,361 cases and 43,923 controls (15). And summary statistics for pre-eclampsia or eclampsia were sourced from the Finn biobank which contained 3,903 cases and 114,735 controls. The threshold for SNP selection was P < 5e–8 and all SNPs and related data were sourced from studies that separately analyzed only populations of European ancestry to eliminate demographic stratification bias.

### IVs selection

The linkage disequilibrium (LD) in selected SNPs was tested to ensure the data were valid. We conducted the clumping procedure to filter independent SNPs within a window size of, 5000kb and r2<0.01 threshold. Then, the F-statistics were calculated to evaluate the strength of each IV and exclude weak instruments. The following rigorous mathematical formula was adopted:


F=R2×(N−K−1)/[K×(1−R2)]

where R2 indicates exposure variance explained by each IV, N refers to the sample size of the GWAS, and K denotes the number of SNP for MR analysis. F>10 indicated sufficient strength of the instruments, which meant the IVs had enough estimated effect for the subsequent MR analysis without weak-tool bias. In this study, we used 65 SNPs as eligible IVs after eliminating ten SNPs with minor allele frequency (MAF) less than the threshold of 0.01. Finally, according to alleles and allele frequencies, we harmonized the SNPs of exposure and outcome by removing or adjusting SNPs with inconsistent alleles to ensure they have corresponding alleles.

### Statistical analyses

In this study, we used the inverse variance weighting (IVW) method as the main approach to deduce the potential causal relationships between RA and pre-eclampsia. Other four effective methods including MR-Egger, weighted median, weighted mode, and Simple mode were also applied to evaluate the possible relationship comprehensively.

Characterized by using the inverse of outcome variance as weight and not taking the intercept into account, the IVW method can provide unbiased causality estimates in an ideal state where all selected genetic variations are assumed to be valid IVs without pleiotropy (16). In terms of MR-Egger, although the significant influence of outlying genetic variables may contribute to its low statistical ability, it can infer the corrected causal effect and provide estimates without bias, even if all selected IVs are not valid (16, 17). If at least 50% of the information from valid instruments is accessible, the weighted median method can offer accurate and robust effect estimates (18). And as for the weighted mode, it is reliable on the condition that the largest subset of instruments with similar causal effects is valid.

### Sensitivity analyses

Firstly, several sensitivity analyses were conducted to assess pleiotropy. We performed the mendelian randomization pleiotropy residual sum and outlier (MR-PRESSO) to detect potential outlier variants (19). Furthermore, the MR-Egger regression was used to evaluate the bias generated by gene pleiotropy, of which the intercept is an indicator (17). Secondly, the Cochrane Q statistic was applied to quantify the heterogeneity between SNPs, where the p-value greater than 0.05 indicated no heterogeneity. Thirdly, we utilized the leave‐one‐out analysis to verify where there exist outliers affecting the result strongly by eliminating each SNP in turn and then performing the IVW method on the rest.

At last, scatter and forest plots were provided to visualize the result of the MR analysis. We did all the analyses on the R software (version 4.2.1.; R Foundation for Statistical Computing, 2021) and RStudio(version, 2022.07.0 + 548), and R package TwoSampleMR and MR-PRESSO were used.

## Result

Sixty-five independent (LD R^2^< 0.01) and genome-wide significant (p< 5e-08) SNPs were identified as qualified IVs with all F-statistics above 10. Detailed information is available in Supplementary Data Sheet 1.

**Figure 2:**
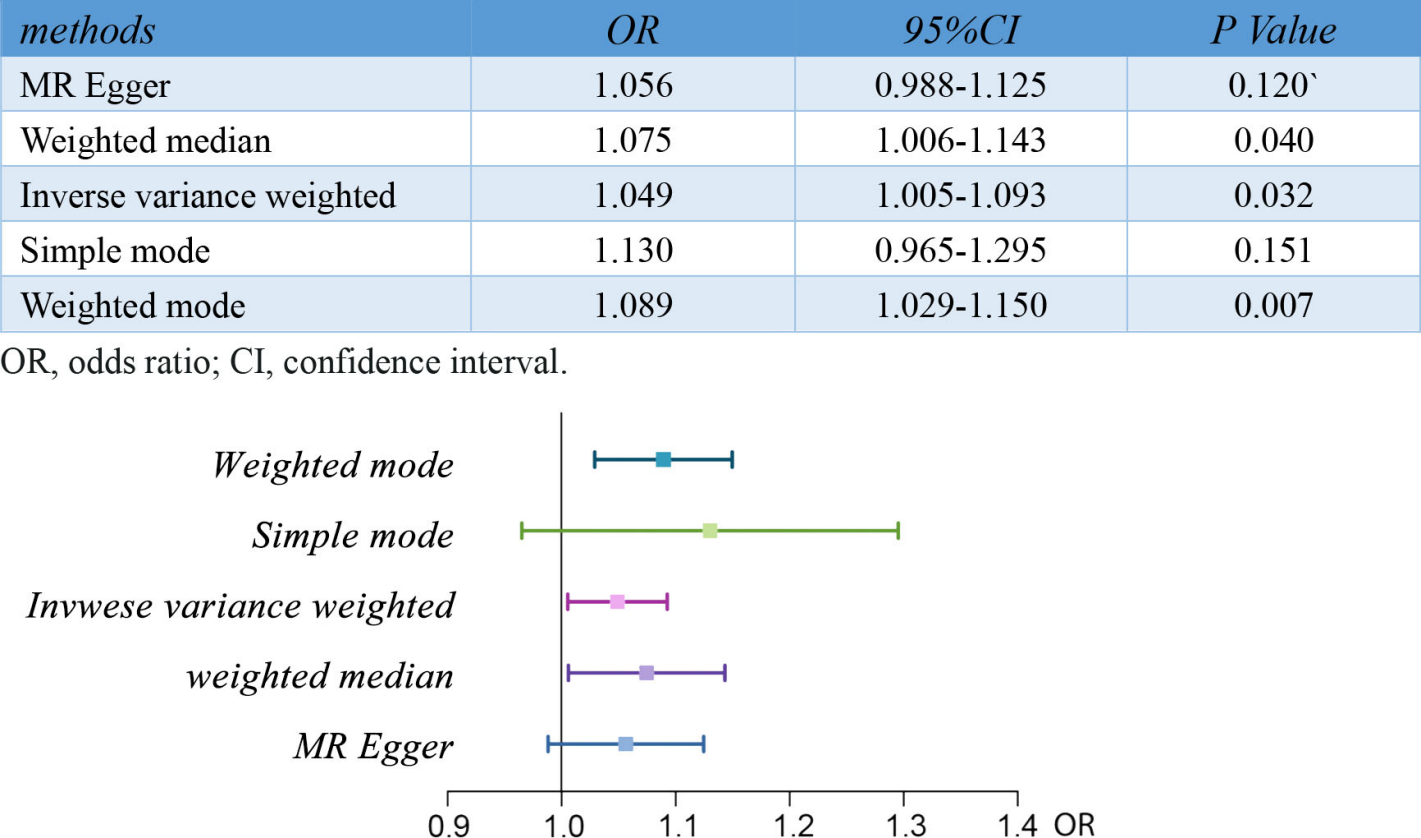
Results of MR analyses conducted to estimate potential associations between RA and risk of pre-eclampsia. MR, mendelian randomization; RA, rheumatoid arthritis.

Notably, the two-sample MR analyses showed a strong causal relationship between RA and pre-eclampsia or eclampsia[OR,1.05;95%CI, 1.01-1.09;p<0.05]. The OR estimates obtained from the weighted mode[OR,1.09;95%CI,1.03-1.15;p<0.01] and weighted median[OR,1.07;95%CI, 1.01-1.14;p<0.05] were similar to those from IVW method, but there was no significant association observed in MR Egger and Simple mode analysis. The results of the five MR analysis methods are shown and visualized in **Figure 2**.

**Figure 3:**
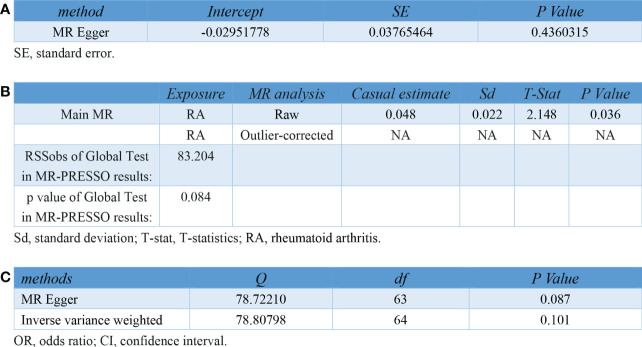
Pleiotropy and heterogeneity testing in sensitivity analyses of IVs for RA. **(A)** Pleiotropy testing using MR Egger regression **(B)** Pleiotropy testing using MR-PRESSO. **(C)** Heterogeneity testing using the Cochrane Q statistic. IV, instrument variant; RA, rheumatoid arthritis; MR, mendelian randomization; MR-PRESSO, mendelian randomization pleiotropy residual sum and outlier.

**Figure 4:**
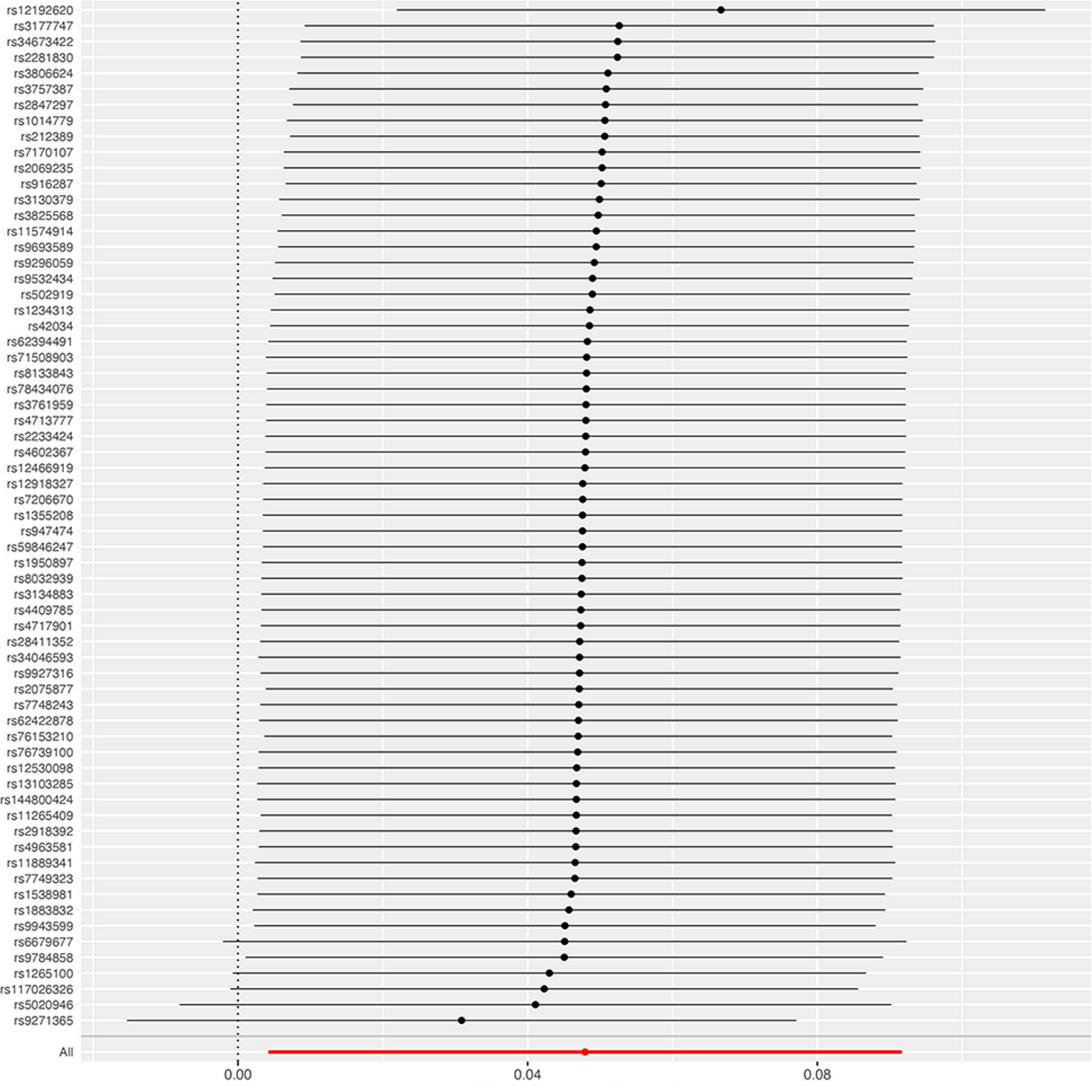
Leave-one-out sensitivity analysis of the causal effect of RA on pre-eclampsia. The red line indicates reliable estimations from the IVW and MR Egger methods. RA, rheumatoid arthritis; IVW, inverse variance weighting.

**Figure 5:**
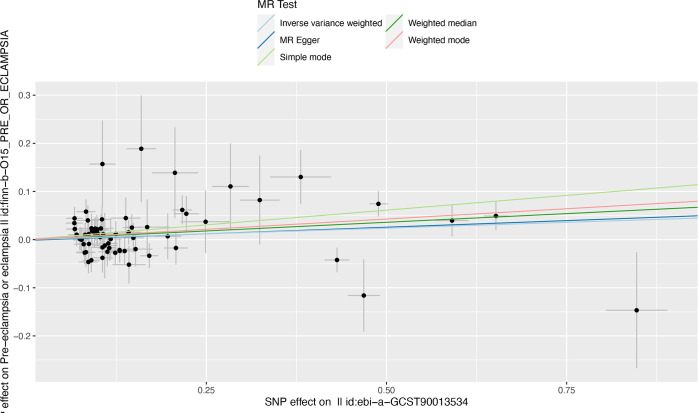
Scatter plot illustrating the distribution of individual ratio estimates of RA with pre-eclampsia as the outcome. Trend lines derived from five different MR methods indicate cause and effect. Inverse variance weighted(light blue), Weighed median(dark green), MR Egger(dark blue), weighted mode(red), Simple mode(light green); SNP, single-nucleotide polymorphism; RA, rheumatoid arthritis; MR, mendelian randomization.

In the sensitivity analyses, no evidence for directional pleiotropy was obtained when we performed MR-Egger regression to reanalyze the result[p=0.79]. And no outlier SNPs were identified by using MR-PRESSO in our study. Heterogeneity was evaluated by Cochrane’s Q test, and the results of both IVW and MR Egger analysis were not significant[p>0.05](**Figure 3**). Furthermore, leave-one-out plots suggested the causal estimates were unlikely to be influenced by certain SNPs (**Figure 4**). In addition, SNP effects individually and jointly from each MR method were displayed in scatter plots (**Figure 5**).

## Discussion

For the first time, we conducted a two-sample MR analysis to investigate the causal effect of RA on pre-eclampsia, which suggested a strong positive association. The results were reliable and robust in sensitivity analyses. We chose the largest genome-wide meta-analysis result for exposure with the advantage of analyzing two distinct ancestral populations originally. Results in the European population could be validated by the other one, which especially provided more reliable GWAS data for MR analysis (15).

Several previous observational studies have been conducted but the conclusions were not consistent. Contradictory to our results, a study with a sample size of 2,802 investigated the risk of pregnancy complications in women with RA in Washington State and showed no significant difference in pre-eclampsia (10). A similar result was revealed when Norwegian researchers conducted a study of 631 women diagnosed with RA before the age of 45, but the exposure of this study included women diagnosed with RA as well as other types of chronic inflammatory arthritides, to some degree, reducing the accuracy of the association inference (20). Due to the limitation of sample size, ethnic homogeneity, and insufficient information, these observational studies with negative results may carry various biases and cannot provide very accurate inferences. On the other hand, our positive causality is in accordance with several previous studies that concluded pregnant women with RA suffered a higher risk of pre-eclampsia. In a retrospective population-based study in the United States, women with RA had a greater likelihood to develop eclampsia or pre-eclampsia. It emphasized the awareness of these risks and suggested women with RA be closely monitored for high-risk pregnancy (21). Not only pre-eclampsia but also other pregnancy complications had a higher frequency to occur when RA patients were pregnant, so doctors should pay more attention to evaluating and detecting complications during the entire pregnancy process (22). In a word, whether RA will increase the risk of pre-eclampsia during pregnancy or not is inclusive, and more reliable analyses should be conducted to provide more solid evidence and give suggestions for clinical practice.

**Figure 6:**
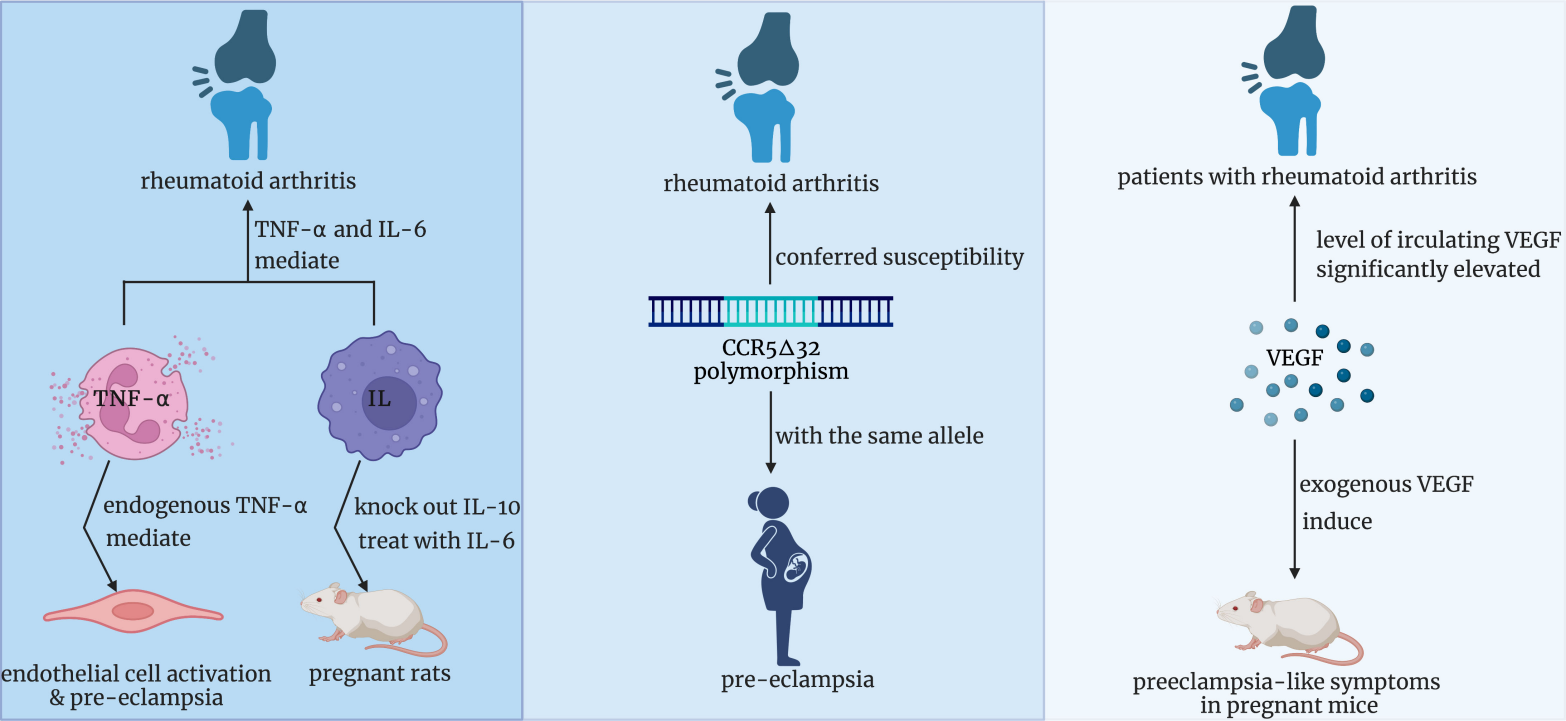
Three potential underlying mechanisms of the positive correlation between RA and pre-eclampsia. RA, rheumatoid arthritis.

The positive correlation between RA and pre-eclampsia has been presumed for multiple reasons (**Figure 6**). Firstly, data from animal studies conducted by Babbette LaMarca et al. supported that endogenous TNF-alpha was vital in mediating endothelial cell activation and pre-eclampsia (23). Meanwhile, Zhongbin Lai et al. and Julia M Orshal et al. respectively examined the crucial role of interleukin-6(IL-6) in the pathogenesis of eclampsia by knocking out the IL-10 gene and treating pregnant rats with IL-6 (24, 25). It is well known that IL-6 and TNF-α are confirmed to mediate many chronic inflammatory diseases, especially rheumatoid arthritis (26, 27). Therefore, it is reasonably assumed that increased production of IL-6 and TNF-α in RA patients might be associated with a higher risk of pre-eclampsia. Secondly, from the molecular perspective, research respectively confirmed that cysteine–cysteine chemokine receptor type 5 (CCR5) delta 32 polymorphism conferred susceptibility to both RA in Europe with a significant negative association and pre-eclampsia in the population consisting of Caucasians with the same allele (28, 29). These two studies provided evidence for the assumption that patients with RA may possess less mutation of CCR5 delta32, which increases the incidence of pre-eclampsia. Notably, animal experiments and further studies are required to confirm this inference. Thirdly, another hypothesis focuses on the role of vascular endothelial growth factor(VEGF). Yusuke Murakami et al. found that exogenous VEGF can induce preeclampsia-like symptoms in pregnant mice (30). Meanwhile, a systematic review and meta-analysis concluded that compared with that in the healthy population, the level of circulating VEGF in patients with RA was significantly elevated (31). So, we presumed that the upregulated expression of VEGF in RA patients might induce pre-eclampsia during pregnancy.

The results of several related research should be considered. Bandoli, G. et al. found that pregnancy complications, particularly pre-eclampsia, can mediate some excess risk for adverse pregnancy outcomes(APOs). Based on the calculation, 20.4% of the excess preterm birth that happened to women with RA was due to pre-eclampsia/hypertension and it also accounted for more than 10% of the excess cesarean delivery (32). Interestingly, according to a systematic review of the risk of APOs prior to the onset of an autoimmune rheumatic disease, the incidence of APOs in pre-RA pregnancies was similar to that in the general population, which suggests a reassuring pregnant condition for pre-RA women (33).

A chief strength of our study is the two-sample MR design which allowed us to evaluate the potential causal effect based on large-scale GWAS with credible sample sizes(14,361 RA cases and 43,923 controls; 3,903 pre-eclampsia/eclampsia cases and 114,735 controls). In the meanwhile, because SNPs are randomly distributed at conception, biases caused by potential confounders and reverse causation can be reduced significantly in this design. It is worth mentioning that our result can present a lifelong pregnant risk for women with RA since genetic variants could not be changed from the time you were born.

The limitations of our study should be noted as well. Firstly, we only enrolled European ancestry populations in our study which means all related data were sourced from studies that separately analyzed only populations of European ancestry. The uniformity of participants eliminates demographic stratification bias and ensures the accuracy of MR analysis results, but whether our findings can be applied to other populations or not remains to be validated. Therefore, further analyses involving other populations require being conducted to confirm whether our findings can be generalized or not. Secondly, the data on outcome was collected from the population with pre-eclampsia or eclampsia and no more specific information was given to describe the severe degree of disease. Further studies are warranted to research the association between RA and subtypes of pre-eclampsia.

## Conclusion

This is the first MR analysis conducted to explore the causality of RA on pre-eclampsia. We find a positive association between RA and elevated risk of pre-eclampsia. Our findings highlight the importance of more intensive prenatal care and early intervention among pregnant women with RA to prevent potential adverse obstetric outcomes. Moreover, our study provides clues for risk factor identification and early prediction of pre-eclampsia.

## Data availability statement

The original contributions presented in the study are included in the article/**Supplementary Material**. Further inquiries can be directed to the corresponding author.

## Author contributions

DZ and SY designed the research. WG and DZ collected and analyzed the data. LY, YL, TO, SY, YZ, and YH performed the literature search. DZ and YH drafted the article. YS, WG, YZ, and YH corrected and edit the article. All authors contributed to the article and approved the submitted version.
